# Structural basis for differentiation between two classes of thiolase: Degradative vs biosynthetic thiolase

**DOI:** 10.1016/j.yjsbx.2019.100018

**Published:** 2020-01-03

**Authors:** Sukritee Bhaskar, David L. Steer, Ruchi Anand, Santosh Panjikar

**Affiliations:** a IITB-Monash Research Academy, Mumbai 400076, Maharashtra, India; b Department of Chemistry, Indian Institute of Technology Bombay, Mumbai 400076, Maharashtra, India; c Department of Biochemistry and Molecular Biology, Monash University, Victoria 3800, Australia; d Monash Proteomics and Metabolomics Facility, Monash University, Victoria 3800, Australia; e Australian Synchrotron, ANSTO, 800 Blackburn Road, Victoria 3168, Australia

**Keywords:** A-mutants, H356A Mutant, AS-mutants, H356A-C90S Mutant, AA-mutants, H356A-C386A Mutant, PcaF, β-ketoadipyl-CoA thiolase, Zr-thiolase, *Zoogleria ramigera thiolase*, Mtb-thiolase, *Mycobacterium tuberculosis thiolase*, Hex-CoA, Hexanoyl CoA, Oct-CoA, Octanoyl CoA, A-mutant-Hex-CoA, A-mutant-Hexanoyl CoA complex, AS-mutant-Oct-CoA, AS-mutant-Octanoyl CoA complex, HAL, hexanal, OAL, octanal, A-mutant-HAL-CoA, A-mutant-hexanal CoA complex, AS-mutant-OAL-CoA, AS-mutant-octanal CoA complex, Covalent locking, Tunnel, Covering loop, Hexanoyl CoA, Octanoyl CoA

## Abstract

Thiolases are a well characterized family of enzymes with two distinct categories: degradative, β-ketoadipyl-CoA thiolases and biosynthetic, acetoacetyl-CoA thiolases. Both classes share an identical catalytic triad but catalyze reactions in opposite directions. Moreover, it is established that in contrast to the biosynthetic thiolases the degradative thiolases can accept substrates with broad chain lengths. Hitherto, no residue or structural pattern has been recognized that might help to discern the two thiolases, here we exploit, a tetrameric degradative thiolase from *Pseudomonas putida* KT2440 annotated as PcaF, as a model system to understand features which distinguishes the two classes using structural studies and bioinformatics analyses. Degradative thiolases have different active site architecture when compared to biosynthetic thiolases, demonstrating the dissimilar chemical nature of the active site architecture. Both thiolases deploy different “anchoring residues” to tether the large Coenzyme A (CoA) or CoA derivatives. Interestingly, the H356 of the catalytic triad in PcaF is directly involved in tethering the CoA/CoA derivatives into the active site and we were able to trap a gridlocked thiolase structure of the H356A mutant, where the CoA was found to be covalently linked to the catalytic cysteine residue, inhibiting the overall reaction. Further, X-ray structures with two long chain CoA derivatives, hexanal-CoA and octanal-CoA helped in delineating the long tunnel of 235 Å^2^ surface area in PcaF and led to identification of a unique covering loop exclusive to degradative thiolases that plays an active role in determining the tunnel length and the nature of the binding substrate.

## Highlights

• Distinguishing degradative and biosynthetic thiolase based on the crystal structures.
• Degradative thiolases have less conserved residues lining the active site.
• Histidine mutation causes covalent locking of the active site in PcaF.
• PcaF contains long tunnel in contrast to Zr-thiolase.
• Pivotal role of the covering loop in distinguishing the two thiolases.

## Graphical abstract

**Figure:**
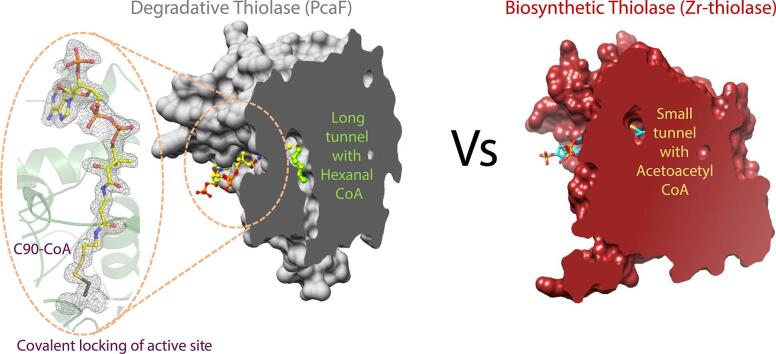

## 1 Introduction

Thiolases, also known as acetyl-coenzyme A acetyltransferases, are a prevalent class of enzymes that are found in both prokaryotes and eukaryotes. The enzymes of this broad class partake in diverse biochemical pathways ranging from fatty acid metabolism to bacterial aromatic compound degradation and are subdivided into two categories; the degradative thiolases and the biosynthetic thiolases (Haapalainen et al., 2006). Degradative or 3-ketoacyl-CoA thiolases are involved in the thiolytic breakdown of β-ketoacyl-CoA to acetyl-CoA and shorter acyl CoA molecule (Haapalainen et al., 2006, Mathieu et al., 1997) and can accommodate long acyl chain substrates. This catalytic cleavage by degradative thiolases is vital as it accounts for the energy production in cells. Biosynthetic or the acetoacetyl-CoA thiolases catalyze the claisen condensation of two acetyl CoA molecules to give longer chain acetoacetyl CoA (Modis and Wierenga, 2000, Davis et al., 1987a). The biosynthetic class prefers short acyl chains of up to 4 carbon atoms, this group predominantly occurs in the fatty acid and polyketide biogenesis (Kursula et al., 2005). Mostly, all thiolases have the ability to catalyze both the biosynthetic and degradative reactions, however, the degradative reaction is thermodynamically favorable. The two classes of thiolases have related sequences and essentially use the same active site residues to perform the relevant reaction, implying a common ancestor (Anbazhagan et al., 2014). Prior bioinformatics studies have shed some light into the origin of thiolases and have helped in classifying unknown thiolase sequences based on signature catalytic loop sequence (Anbazhagan et al., 2014). Furthermore, phylogenetic studies of thiolase superfamily have indicated the ‘ancestral origin’ of archaeal thiolase compared to the other enzymes of the superfamily (Jiang et al., 2008). Evolutionary studies of eukaryotes have shown the proteobacterial origin of eukaryotic thiolases. During the eukaryotic evolution, the loss or gain of sequences in thiolases genes is also shown to dictate the subcellular location of the thiolases as well as determine the biosynthetic and degradative classes of the thiolases (Pereto et al., 2005).

Till date a large body of work has been focused towards deciphering structural function and mechanism of biosynthetic thiolases (Kursula et al., 2005, Kursula et al., 2002, Davis et al., 1987b). However, not much work has been performed on developing perspectives on the functioning of degradative thiolases. Neither efforts to fully identify factors that distinguish the two classes have been entailed. Hence, to develop a broader understanding on degradative thiolases, in this study, a β-ketoadipyl-CoA thiolase (PcaF) from the β-ketoadipate pathway is explored. This enzyme belongs to *Pseudomonas putida* KT2440, which is a well-studied soil bacterium that can assimilate a variety of aromatic compounds. It possesses four major pathways that degrade aromatics; homogentisate pathway (*hmg/fah*/*mai* genes), the phenylacetate pathway (*pha* genes), the catechol (*cat* genes) and the protocatechuate (*pca* genes) pathway (Jiménez et al., 2002, Abril et al., 1989, Yamanashi et al., 2015).

**Fig. 1:**
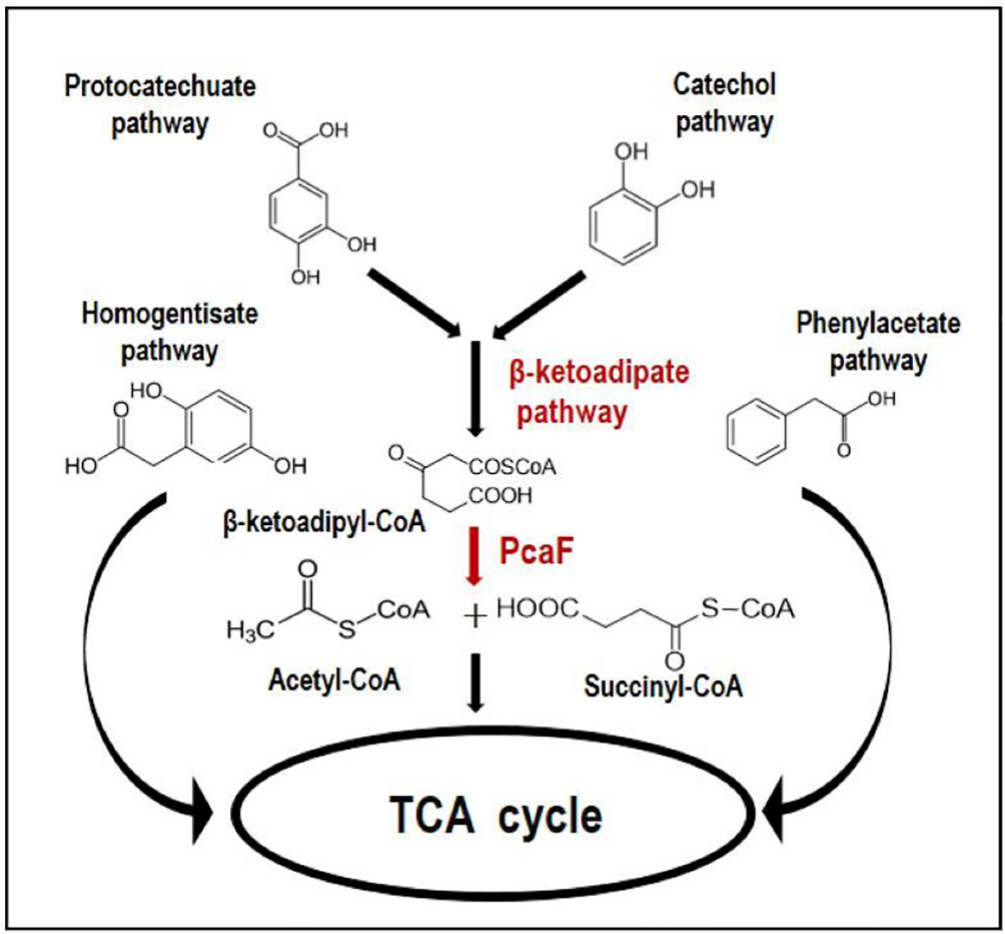
Schematic representation of the microbial aromatic degradation pathways involving protocatechuate, homogentisate, catechol and phenylacetate pathways. The catechol and protocatechuate pathways combine to form the β-ketoadipate pathway, which is preceded by the thiolase PcaF in the last step.

The catechol and protocatechuate pathways converge to form the central β-ketoadipate pathway which is a taxonomically widespread bioremediation pathway in bacteria (Ornston, 1966a, Ornston, 1966b, Ornston, 1966c). In this study, a degradative thiolase, β-ketoadipyl-CoA thiolase (PcaF) from the β-ketoadipate pathway is explored. The *pcaF* gene is located in the gene cluster *pcaRKF* and encodes the last enzyme of the β-ketoadipate pathway involved in the conversion of β-ketoadipyl-CoA into succinyl-CoA and acetyl-CoA (TCA cycle intermediates) (Nelson et al., 2002, Jiménez et al., 2002, Kim et al., 2006). PcaF is a pivotal enzymatic player of the β-ketoadipate pathway as, it concludes the biocatalysis of the toxic compounds from catechol and protocatechuate pathways into non-lethal metabolic intermediates, which enter the TCA cycle (Fig. 1) and aids in energy production in the bacteria.

To understand the active site architecture of the degradative thiolases, single and double active site mutants of PcaF (H356A, H356A-C90S, H356A-C386A mutants referred in the manuscript as A-mutant, AS-mutant and AA-mutant respectively) were made and crystal structures of the apo PcaF as well as the active site variants in-complex with CoA, Hexanoyl CoA (Hex-CoA) and Octanoyl CoA (Oct-CoA) were solved. In A-mutant complex with CoA and A-mutant complex with Hex-CoA complexes, CoA was found to be covalently linked to the active site C90 residue rendering the enzymes’ active site to be blocked. Further, bioinformatics analyses were performed and together the information obtained was vital in understanding the unique features of degradative thiolases. For instance, it was found that both a flexible active site architecture as well as a long tunnel in PcaF renders it capable of accommodating longer chain acyl CoA derivatives. The work encompasses in-depth structural and evolutionary analyses and provides key structural evidences that differentiate degradative verse biosynthetic group of thiolases.

## 2 Results

### 2.1 Overall structure and the active site architecture

**Fig. 2:**
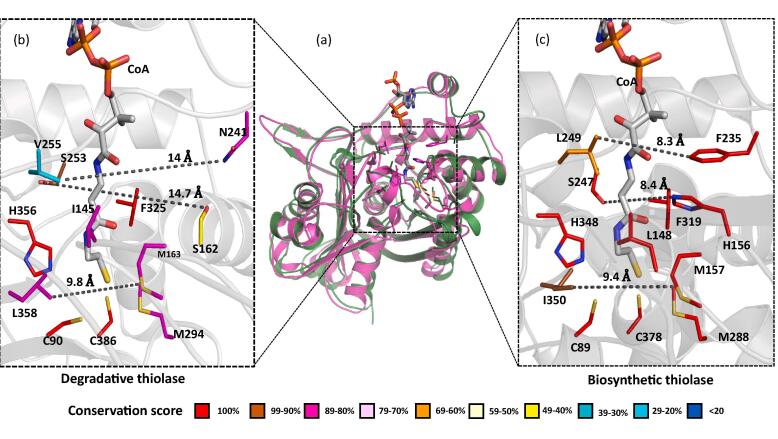
Comparison of active site cavity of a representative member of the thiolase superfamily. Superimposition of the PcaF (in green) and Zr-thiolase (PDB code: 1DLV in magenta) indicate a variation in the active site architecture. The active site architecture of the both degradative (inset B) and biosynthetic thiolase (inset C). Residues are colored according to the conservation score. The conservation score is calculated using 100 sequences of degradative and of biosynthetic thiolases respectively.

The 1.81 Å apo PcaF structure was solved by the molecular replacement (MR) method using a putative acetyl-CoA acetyltransferase (PDB code: 1ULQ, 52% sequence identity). The asymmetric crystallographic unit contains four identical monomers with solvent content 48.45%. The four molecules of PcaF in an asymmetric unit forms a tetramer with an extensive buried surface area of 21130 Å^2^ which is 2.5 time larger than that of PcaF dimer as calculated using PISA (Protein interfaces, surfaces and assemblies, Krissinel and Henrick, 2007). The tetrameric oligomeric state has been seen in a number of other thiolases (Ithayaraja et al., 2016, Pye et al., 2010). The monomers B and C have a continuous density for residues −2 to 400 unlike A and D subunits that lack density for residues 212–215. Each monomer is comprised of the N-domain (residues 1–119 and 256–278), the C-domain (residues 279–400) and the loop-domain (residues 120–255). The overall structural assembly of PcaF monomer comprises of an α/β topology β1α1β2α2β3α3β4β5 together forming a characteristic five layered thiolase superfamily arrangement (Fig. 2), (Haapalainen et al., 2006, Mathieu et al., 1994, Mathieu et al., 1997). Active site residues (Cys90, His356 and Cys386) of PcaF is identical to that of the catalytic triad of biosynthetic thiolases.

One of the interesting questions posed was, how the same set of active site residues catalyze both biosynthetic and degradative reactions. To discern as a representative case, structural analysis of the degradative thiolase PcaF with the biosynthetic thiolase *Zoogleria ramigera* (Zr-thiolase) (PDB code: 1DLU) was conducted. PcaF active site architecture comprises of N241, 325F, S162, M163, M294, S253, V255, H356 and I145 (Fig. 2). These residues were compared across the degradative thiolases. Surprisingly, a sequence alignment using degradative and biosynthetic thiolases showed less than 95% homology. It was noted while the catalytic triad and F325 are fully conserved, M163, M294, S253 and L358 have consensus in the range of 80 to 96%, whereas S162 and V255 are highly variable (Fig. 2).

On further comparison of the native PcaF and Zr-thiolase, it is observed that the Zr-thiolase has an equivalent active site architectures, flanked by residues*,* F235, F319, H156, M157 and M288 on one side and on the other side by S247, L249, H348, L148 and I350 (Modis and Wierenga, 2000) (Fig. 2, left wall) on the other side. V255, I145, N 241, S162 and L358 are analogous residues in PcaF that form the left CoA pocket lining and corresponding L249, L148, F235, H156 and I350 residues line the other side. The residues comprising the active site architecture in Zr-thiolase are fully conserved across biosynthetic thiolase sequences, except for L249 and I350 residues which are 68% and 96% conserved. Comparison of the active site walls reveal that even though the catalytic triad is conserved in the degradative and the biosynthetic thiolases, the overall active site lining is different (Fig. 2). The biosynthetic active site is well-demarcated and highly conserved compared to the degradative thiolase which has more flexibility in active site residues. The rigidity observed corroborates with the structural profile of the biosynthetic group which is limited to short four carbon chain acyl CoA and lacks the versatility and range exhibited by degradative thiolases. As can be seen in Fig. 2 the active site of degradative thiolases is wider and is adaptable to accommodate a wider substrate variety.

### 2.2 Native and mutant ligand complexes

The kinetic parameters of native PcaF were measured and K_m_ (67.8 ± 8.2 µM) and k_cat_ (0.7 ± 0.08 S^−1^) for acetoacetyl CoA were observed. Single (H356A: A-mutant) and double active site mutations (H356A-C90S: AS mutant, and H356A-C386A: AA-mutant) were cloned and purified (see materials and methods). The expectation was that these mutants will be inactive therefore, capturing ligand-binding complexes will be easier to undertake. Activity measurements of A-, AS- and AA-mutants revealed that these mutants exhibited 30%, 7% and 5% activity respectively, relative to native PcaF. Due to substantial reduction of activity these three mutants therefore, turned out to be suitable candidates for ligand binding and for trapping a wide range of ligands. β-Ketoadipyl CoA is the natural substrate of PcaF which is not commercially available. β-Ketoadipyl CoA belong to the last step of aromatic degradation pathway and crystallographic studies conducted by using the natural substrate would have provided further insight into the its interaction with PcaF. Moreover, β-Ketoadipyl CoA would have been a biologically relevant substrate for determining the kinetic parameters of PcaF. Nonetheless, the closest analogue to β-Ketoadipyl CoA is the six-carbon acyl Hex-CoA. The crystals of the A-mutant, AS-mutant and AA-mutant proteins were soaked for 20 to 300s with Hex-CoA and other accessible varieties of CoA derivatives (CoA, Acetyl CoA, Acetoacetyl CoA, Oct-CoA, and Decanoyl CoA) as the molecular packing density (Fig. S1) suggested that the diffusion of varieties of CoA derivatives into the PcaF crystals might be possible without the need of co-crystallisation.

Altogether 18 datasets were acquired in the resolution range of 2.56 to 1.37 Å and analysed in detail.

In the A-mutant and AA-mutant, it was observed that the CoA and the other longer chain fatty acyl CoAs form a disulfide bond with the C90 active site residue. As a consequence, in the higher chain-length CoAs after the -SH atom there is a cleavage to form a covalently linked C90-SCoA adduct, the remaining acyl chain of CoAs exist in the aldehyde form. In the case of AS-mutant, the cleavage after–SH atom also occurred but no disulfide bond was formed. For ligands acetyl CoA, acetoacetyl CoA and decanoyl CoA, only the CoA part was trapped in all the mutants and the missing acyl parts seemed to be released from the protein. Only in A-mutant-Hexanoyl CoA complex (A-mutant-Hex-CoA) and AS-mutant-Octanoyl CoA (AS-mutant-Oct-CoA), both the CoA and the cleaved acyl part are observed and thus a total of four structures scenarios are reported in this study; the apo PcaF, A-mutant-CoA complex, A-mutant-Hex-CoA complex and AS-mutant-Oct-CoA (Table 1). The real space correlation co-efficient (RSCC) values for the ligands present in A-mutant-CoA, A-mutant-Hex-CoA and AS-mutant-Oct-CoA complexes are shown in Table S1, Table S2 and Table S3 respectively.

**Table 1:** Data-collection, processing and refinement statistics.

Dataset	PcaF	PcaF, H356A-CoA complex	PcaF, H356A-Hex-CoA complex	PcaF, H356A-C90S-Oct-CoA complex
Protein/complex	*(apo)*	*(A-mutant complexed with CoA)*	*(A-mutant complexed with Hex-CoA)*	*(AS-mutant complexed with Oct-CoA)*
*Unit cell data*				
Space group	P2_1_2_1_2_1_	P2_1_2_1_2_1_	P2_1_2_1_2_1_	P2_1_2_1_2_1_
Number of subunits in the asymmetric unit	4	4	4	4
Unit-cell parameters(Å, °)	*a* = 110.08, *b* = 113.84, *c* = 127.42, α = β = γ = 90	*a* = 110.49, *b* = 115.96, *c* = 128.87 *α = β = γ =* 90	*a* = 111.39, *b* = 116.40, *c* = 127.90, *α = β = γ =* 90	*a* = 111.08, *b* = 116.62, *c* = 128.56 α = β = γ = 90
Dordrecht				
Beam line	Australian Synchrotron	Australian Synchrotron	Australian Synchrotron	Australian Synchrotron
Wavelength (Å)	0.9537	0.9537	0.9537	0.9537
Resolution (Å)^a^	20–1.81 (1.92–1.81)	20–1.61 (1.70–1.61)	20–1.96 (2.08–1.96	20–1.37 (1.45–1.37)
Observed reflections^a^	998,852 (158022)	1,445,863 (218460)	697,289 (106899)	2,371,592 (356212)
Unique reflections^a^	281,317 (44875)	413,663 (65788)	220,195 (35163)	677,103 (108090)
Data completeness (%)^a^	99.5 (98.1)	99.3 (97.6)	95.8 (94.7)	99.5 (98.1)
<I/σ(I)>^a^	10.28 (2.18)	11.36 (1.85)	8.52 (2.42)	11.96 (2.09)
Multiplicity^a^	3.55 (3.52)	3.49 (3.32)	3.16 (3.04)	3.5 (3.29)
R_merge_ (%)^a^	8.8 (59.6)	6.8 (56.0)	11.3 (51.7)	6.3 (55.0)
R_meas_ (%)^a^	10.4(70.2)	8.1(66.9)	13.6 (62.4)	7.4(65.6)
CC_1/2_ (%)^a^	99.7 (69.6)	99.8 (69.5)	99.2 (74.2)	99.8 (71.0)
Wilson *B* factor (Å^2^)	19.2	17.8	19.0	12.4
***Refinement***				
R_work_ (%)	15.59	16.07	17.58	14.00
R_free_ (%)	19.58	19.61	20.81	17.20
*Number. of atoms*				
Protein atoms	11,799	11,850	11,813	11,982
Ligand atoms	64	270	286	147
Solvent atoms	995	1324	1216	1835
***Model quality***				
*RMS deviation from ideal value*				
Bond length (Å)	0.009	0.009	0.007	0.008
Water Bond angle (°)	1.562	1.606	1.493	1.688
Average B-factor				
Protein atoms (A/B/C/D) (Å^2^)	20.1/20.7/ 21.2/20.3	23.4/22.1/ 20.5/21.1	22.7/22.3/ 19.7/19.4	21.9/20.1/ 18.3/18.9
Ligand atoms (Å^2^)	27.5	30.2	32.2	35.0
Waters (Å^2^)	30.1	30.9	28.1	35.3
*Ramachandran plot*^b^				
Most favored regions (%)	96.5	96.8	95.6	97.0
Allowed regions (%)	3.1	2.8	4.0	3.0
Outlier regions (%)	0.4	0.4	0.4	0.0
PDB code entry	6PCA	6PCB	6PCC	6PCD

a Values in parentheses refer to the highest resolution shell.

b Calculated by MOLPROBITY (Chen et al., 2010).

### 2.3 Covalent locking of the active site

**Fig. 3:**
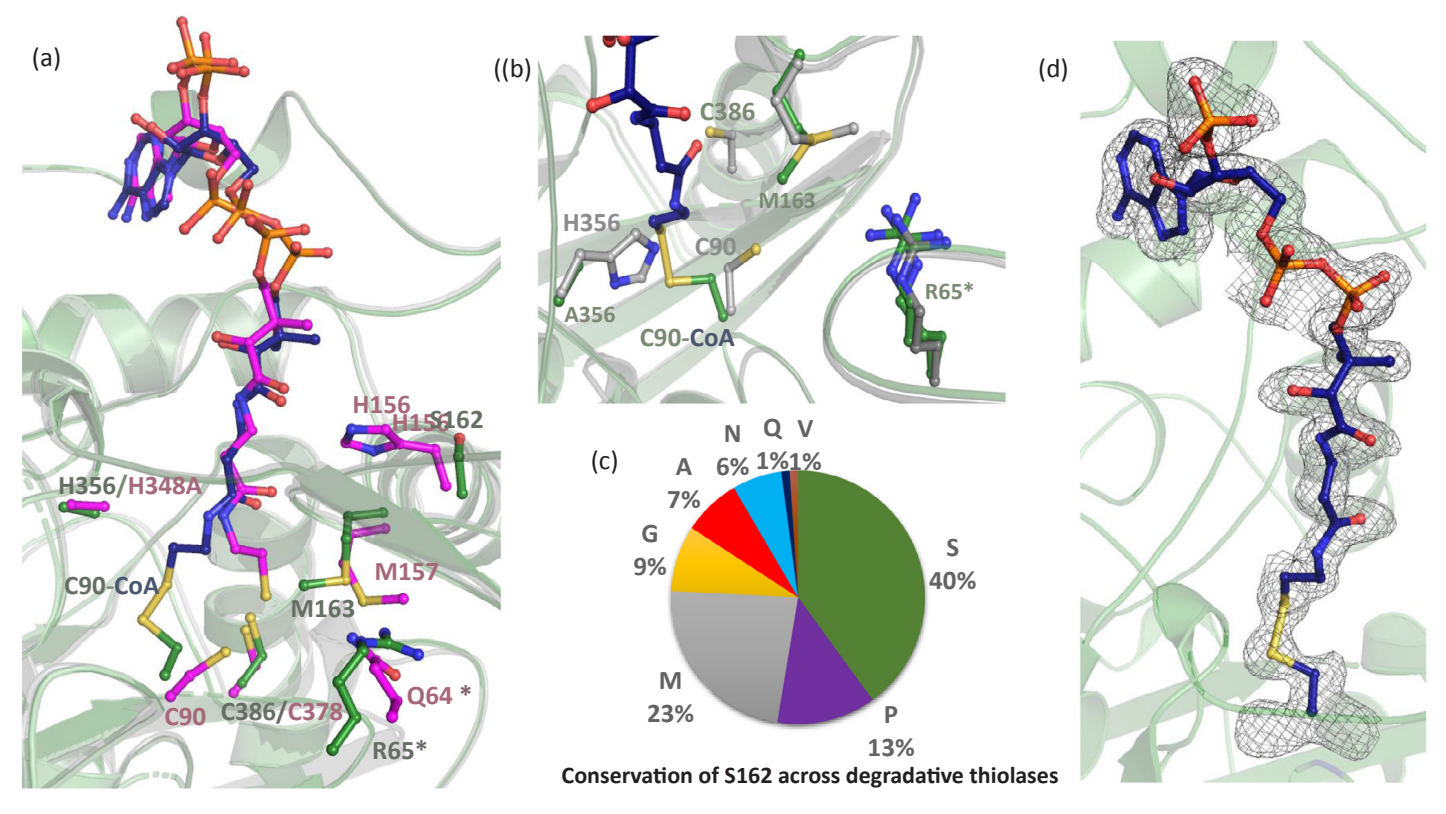
Analysis of the covalent locking of the active site in PcaF (a) Superposition of A-mutant − CoA covalent complex of PcaF (the protein is in green and the covalently bound CoA is colored in blue) with H348A mutant-CoA complex of Zr-thiolase (PDB code: 2WL4, magenta). R65 and Q64 residues marked with “*” are from the adjacent subunit. (b) Superposition of native PcaF and A-mutant − CoA covalent complex of PcaF colored in grey and green respectively. (c) The pie chart shows the conservation of S162 equivalent position across 100 degradative thiolases. (d) Simulate annealing omit map contoured at 3σ highlighting the covalent linkage between C90 and CoA in A-mutant.

As stated above structural analysis of the A- and AA-mutants complexed with CoA reveals a disulfide bond formation between the C90 active site residue and the thiol group (–SH atom) of the CoA leading to covalent blocking of the active site (Fig. 3a and d). This is an uncustomary thiolase reaction that is not previously reported. It seems that the H356A mutation (A-mutant) compromised the activity of the PcaF leading to a locking of the reaction center (Fig. 3). In the case of the AS-mutation, the disulfide bond was not detected as a serine is present instead of cysteine. However, the thiol group of CoA is found in a double conformation (Fig. S2).

Similar mutation in the crystal structure of the Zr-thiolase was reported by Meriläinen *et al.* (PDB code: 2WL4), where the H348A (equivalent to the H356 position in PcaF) was mutated. However, in Zr-thiolase mutant complexed with CoA without covalent-C89 (corresponding to C90 position in PcaF) was observed (Meriläinen et al., 2009). Therefore, the question arises, why identical mutation of the active site histidine residue in the two types of thiolases yields different binding modes. Analysis of the superimposed A-mutant-CoA complex of PcaF and Zr-thiolase (PDB code: 2WL4) structures indicates that the presence of S162-M163-P164 (SMP) motif near the active site cavity in the degradative thiolase PcaF which corresponds to conserved H156-M157-G158 (HMG) motif of the Zr-thiolase may be responsible for the difference. The triad found in degradative thiolase appears to form a rigid architecture forcing covalent bond formation. Whereas, in Zr-thiolase this region looks to be flexible and can move to prevent the formation of this deadlock complex. Another potential difference is the occurrence of R65 residue in the degradative thiolase PcaF which corresponds to Q64 in the Zr-thiolase, (both residues are adjacent monomeric subunit of the dimer. H156 is a fully conserved residue across the biosynthetic thiolases and likely act as an “anchoring” residue by mediating interaction with the phosphopantetheine moiety of CoA and holds the substrates in the correct conformation (Fig. 3a). This is because both the CoA and acyl CoAs are large moieties that require such an anchoring point for the active site activity. In degradative thiolases, the equivalent position is occupied by a variety of residues (Fig. 3c), indicating the lesser conservation of the said position. In the case of PcaF, it is occupied by S162 where the residue is flipped away from the active site cavity and consequently cannot act as an anchor (Fig. 3a). Instead, it appears that the H356 residue of the catalytic triad acts as an anchoring residue. Thus, when the H356 residue is mutated to alanine, the tethering point for the substrate is lost and the thiol bond of CoA exposed to the active site. This allows residue C90 to attack other positions instead of the usual Cα and Cβ of 3-ketoacyl CoA. The H356A mutation also causes changes in the neighboring residues M163’ and R65’ (from another monomer of the dimer in PcaF). In the apo structure, M163 is located 6.72Å (CE-SG distance) away from C90 and 3.75Å (CE-NH2 distance) away from R65, whereas in A-mutant-CoA complex structure, the side chain of M163 moves 3.78Å away from its native conformation which is toward the active site cavity and this allows the R65 residue to undertake in double conformations (Fig. 3b). Interestingly, side chain of the M157 residue in the biosynthetic thiolase (PDB code: 2WL4), equivalent to the M163 residue of the A-mutant-CoA complex adopts similar conformation to that of in the native PcaF (Fig. 3a and b). The varied anchoring sites, high conservation of the active site architecture in the biosynthetic and the additional role of H356 in the degradative thiolase PcaF emphasizes the difference between the two classes of thiolases.

### 2.4 Mass spectroscopic analysis of covalent locking of the active site

Intact mass spectrometry was carried out on two set of proteins; the apo protein and the AA-mutant. We have chosen AA-mutant to perform mass spectrometry in presence of CoA derivatives to show that the covalent locking occurs due to the C90 only and not due to the other cysteine (C386) at the active site. The apo PcaF comprises of 400 amino acids along with an additional 22 amino acids from the vector and its molecular weight (MW) is 44454.91 Da, whereas MW of the AA-mutant is 44356.79 Da.

The observed mass spectrometry peak for apo protein and for AA-mutant in the absence of any CoA derivative was 44376.3 Da (Fig. S3a) and 44343.6 Da (Fig. S4a) respectively. There are discrepancies of 78.61 Da less and 13.19 Da more in the measured molecular weight of apo and AA mutant proteins respectively, compared with the calculated values. Such differences in the measurement are due to unknown modifications and is therefore critical to compare experimental values of the control with the modified sample and not using calculated values when determining any changes made to a protein.

The mass spectrometry was carried on the apo protein in presence of acetoacetyl CoA and the same observed peak (44376.3 Da) was detected as for the apo protein alone (Fig. S3b) indicating that the native protein did not undergo covalent locking of CoA (Table 2).

**Table 2:** Intact mass spectrometry studies using apo protein and AA mutant*.*

Samples	Observed mass	Modification observed
Control apo protein	44376.3 Da	–
apo-ligand complex (apo protein-acetoacetyl CoA)	44376.3 Da	No
Control AA-mutant	44343.6 Da	–
AA-mutant-ligand complex (AA-acetoacetyl CoA)	45110.8 Da	Yes
AA-mutant-ligand complex (AA-hexanoyl CoA)	45110.4 Da	Yes
AA-mutant-ligand complex (AA-octanoyl CoA)	45110.4 Da	Yes

The observed mass spec peak for the AA mutant in presence of acetoacetyl CoA, hexanoyl CoA and AA-octanoyl CoA were 45110.8 Da, 45110.4 Da and 45110.4 Da indicating increases of 767.2 Da, 766.8 Da and 766.8 Da respectively relative to the observed mass peak of AA mutant (Fig. S4). The calculated molecular weight of CoA is 767.5 Da and the observed molecular weight of the modification for the AA-mutant-ligand complexes were ~ 767 Da. Thus, the intact mass spectrometry confirmed the covalent modification of the AA-mutant and the similar observation has been made for A-mutant (data is not shown).

### 2.5 Radiation damage analysis

A-mutant complex with hexanoyl CoA dataset and the refined crystal structure were used for radiation damage analysis. The isomorphous difference Fourier map (model phase, F_before_ – F_after_) was calculated as described in Material and Method section. The analysis of the difference Fourier map provides 20 peaks above 5σ and highest peak being 6.8σ. The majority of the peaks are near the Glu and Asp residues indicating decarboxylation due to radiation damage (Fig. S5). Interestingly, significant peak was found at Sγ atom of Cys-90 from chain B and C as well as at S1P atom of CoA molecules, which are covalently bound. This indicates that the co-valent bond in chain B and C has suffered from the X-ray radiation damage (Fig. S5b). However, there was no trace of radiation damage at the oxygen atom of the hexanal (Fig. S5b). Similarly, in isomorphous difference Fourier map from A-mutant-CoA complex dataset, with peak height 5.6 σ–6.2σ were found at Sγ atom of Cys-90 from chain A, B and C and 8.1σ–9.0σ at S1P atom of CoA molecules in all chains (Fig. S5a), other significant peaks were near the Glu and Asp residues and sulfur atom of the methionine residues.

### 2.6 Longer chain CoA binding tunnel

The degradative thiolases have the ability to catalyze longer chain substrates compared to the biosynthetic thiolases (Feigenbaum and Schulz, 1975). Accordingly, to accommodate the longer aliphatic chains it is expected that they have evolved to harbor long active site tunnel to bind the reactant. As hypothesized in the degradative thiolase PcaF, an interconnected network of tunnels with a long continuous channel running across the dimer is revealed using the program CAVER (Fig. S6a). In the past several computational studies on these thiolase based systems have suggested that the substrate binding sites usually comprise of a large tunnel/ concavity in the proteins (Byska et al., 2016). Here, using structure we first locate the tunnels. Thus, the tunnel sizes of a single monomer (monomer-A) were independently calculated using CASTp (Tian et al., 2018) wherein two major tunnels were observed with surface area 154 Å^2^ and 235 Å^2^ respectively. The remaining tunnels have surface area less than 53 Å^2^, which are too small to form an active site tunnel. The first tunnel with 154 Å^2^ surface area, corresponds to the tetramerization loop region. The second tunnel with 235 Å^2^ surface area is located near the covering loop (Fig. S6b). Involvement of a covering loop in substrate binding have been proposed in the previous studies (Kiema et al., 2014, Harijan et al., 2017). In PcaF, the covering loop is formed by the loop-domain residues ranging from 143 to 163. The second tunnel located close to the covering loop is the substrate binding site that is validated by the ligand bound structure wherein the acyl tail of the substrate binds.

**Fig. 4:**
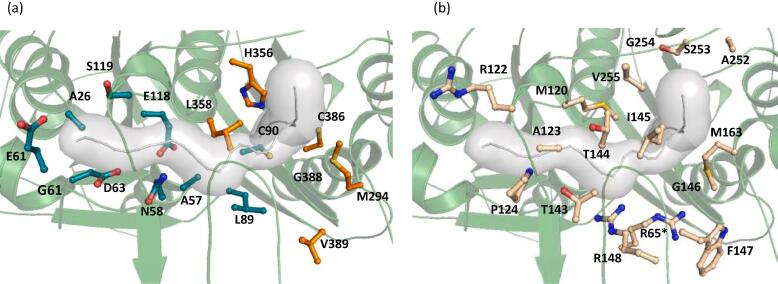
The residues partaking in tunnel formation are highlighted. (a) Residues involved in forming the tunnel from N- and C-terminal domain are colored deep teal and orange respectively. (b) The residues from the loop-domain contributing to the tunnel formation are in light yellow.

An in-depth analysis of the tunnel residues reveals that the residues participating in the tunnel formation are a combination of hydrophobic and hydrophilic amino acids. The key residues observed in the tunnel are shown in Fig. 4. Residues from both the N -terminal and C-terminal domain constitute the tunnel. While the back end of the tunnel is formed by N-terminal residues whereas the front-end is formed by the C-terminal residues (Fig. 4a). Majority of the tunnel residues belong to the loop-domain (Fig. 4b). Residues T143-G146 and R148 contribute in tunnel formation belong to the covering loop. Contrastingly, in Zr-thiolase (PDB code: 1DLU) only one tunnel (with surface area of 132 Å^2^) was observed. Upon comparison Zr-thiolase tunnel with the equivalent tunnel in PcaF, it is observed that the surface area of the biosynthetic thiolase tunnel is almost half that of the degradative thiolase tunnel. This implies that PcaF being a degradative thiolase can accommodate longer chain substrates and is equipped with a larger active site tunnel (Fig. S7).

### 2.7 Promiscuity of the degradative thiolase

**Fig. 5:**
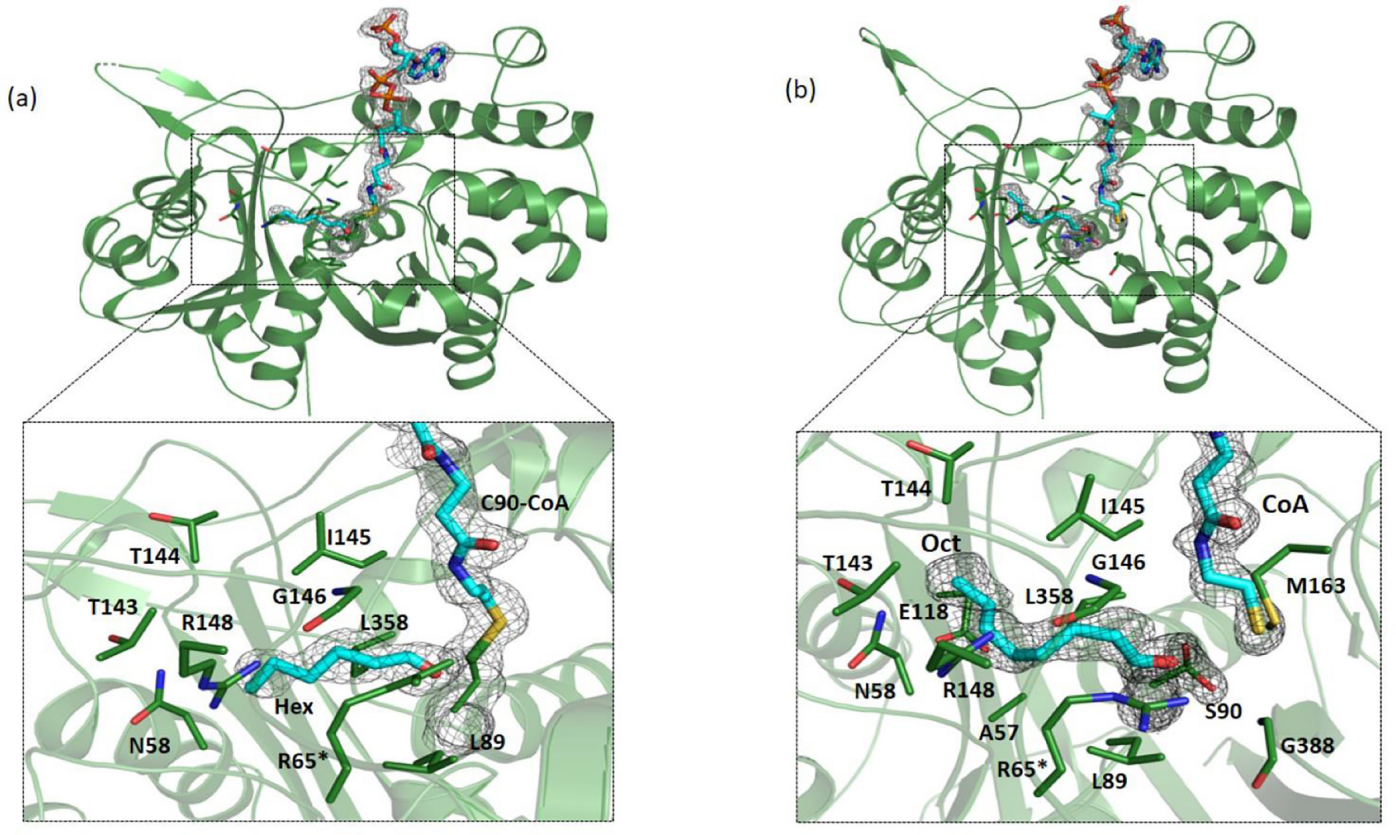
Representation of substrate binding in the long tunnel of PcaF. The interaction of the tunnel residues (a) with the hexanal part of hexanoyl CoA-mutant complex (b) with the octanal part of octanoyl CoA-AS mutant complex of PcaF. Carbon atoms of the ligand binding residues are shown in green and the ligands are shown in cyan. R65* indicates the residue from the adjacent subunit. Simulate annealing omit maps for the ligands are contoured at 3σ.

The A-mutant and AS-mutant when soaked with 3 mM Hex-CoA and Oct-CoA, respectively, led to two complexes. The A-mutant-Hex-CoA complex contains the C90 that is covalently linked to CoA and the cleaved hexanoyl derivative in the form of hexanal (HAL). The AS-mutant-Oct-CoA complex comprises of cleaved Oct-CoA in which the CoA remains free and the cleaved octanoyl derivative in the form of octanal (OAL). None of the previous degradative thiolase structures have been obtained in complexed with any longer chain CoA derivatives. Here, we were able to trap both C_6_ and C_8_ acyl CoA chain bound to PcaF, A-mutant-hexanal CoA (A-mutant-HAL-CoA) and AS-mutant-octanal CoA (AS-mutant-OAL-CoA) (Fig. 5). These structures provide insight into binding of the long chain substrates and helped in identifying the novel tunnel which accommodates longer tail of the substrates. The structures also aid in delineating the residues that partake in interacting with the acyl tail and in understanding the adaptability of the degradative thiolase to bind to a variety of substrates.

HAL and OAL moieties are stabilized in the proposed tunnel via various hydrophobic interactions (Fig. 5). In fact, there is more space is remaining in the tunnel after accommodating the acyl chains. The calculated surface area of the tunnel is 235 Å^2^ and the surface areas of the HAL and OAL are 124 Å^2^ and 152 Å^2^, respectively. An extra 83 Å^2^ surface area is still available after the tunnel is occupied by the OAL acyl chain which implies that PcaF can bind to the substrates longer than C_8_ acyl CoA. As previously mentioned, the acetyl CoA, acetoacetyl CoA and decanoyl CoA ligands were also used for soaking, however the X-ray data analysis showed that only the CoA moiety of acetyl CoA, acetoacetyl CoA and decanoyl CoA were bound in all the mutants. Since the acyl tail of these longer ligands were missing, it indicates that the soaking time (20–300 s) might have been too long, given that each of these mutants are still active. HAL was found in all four monomeric units and the binding of the cleaved CoA from Hex-CoA is similar to that of the A-mutant-CoA. The tunnel residues that are engaged in interaction with the acyl part of HAL, are shown in the Fig. 5a. In AS-mutant-Oct-CoA structure, OAL was bound in the tunnel of all monomers except in the C-monomer where both CoA and acyl group were missing, and that position was occupied by a glycerol molecule. This may be due to the fact that the AS-mutant-Oct CoA complex is obtained by quick soaking (40 s) and this duration may have been too short for the ligand to bind to all four monomers. Tunnel residues involved interacting with the OAL acyl fragment are depicted (Fig. 5b). Interestingly, both the HAL and OAL moieties interact with the T143- G146 and R148 tunnel residues which are part of the covering loop. 20 amino acids are involved in tunnel formation (Fig. 4b), but only the covering loop forms the backbone of the tunnel and plays a key role in acyl moiety binding.

### 2.8 Significance of the covering loop

**Fig. 6:**
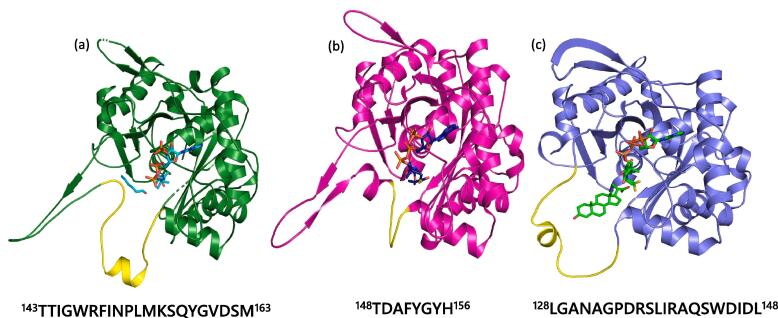
Comparison of the covering loop from representative members of the thiolase superfamily, shown in the same orientation. (a) A-mutant of PcaF complexed with non-natural substrate hexanal and CoA (b) Zr-thiolase complexed with acetoacetyl CoA (PDB code: 1M1O). (c) Mtb-thiolase complexed with steroid-CoA (PBD code: 4UBT). For each structure, the covering loop is colored in yellow and amino acid sequence with numbering of the loop are shown.

As discussed above, the covering loop residues are involved in tunnel formation and also interact with the acyl tail of the longer CoA derivatives in PcaF. To understand the significance of the covering loop across degradative and biosynthetic thiolases, covering loop from three structures; the Zr-thiolase in complex with acetoacetyl CoA (PDB code: 1M1O) (Harijan et al., 2017), the degradative thiolases *Mycobacterium tuberculosis* (Mtb-thiolase) in complex with steroid and CoA (PBD code: 4UBT) (Schaefer et al., 2015) and PcaF A-mutant in complex with hex and CoA were compared (Fig. 6) The three structures superimpose with a root mean square deviation (RMSD) values ranging from 1.36 to 1.42 Å for 366–370 Cα pairs and excluding the covering loop, RMSD values range between 1.21 and 1.32 Å for 356–366 Cα pairs. The decrease in the RMSD indicates a large variation of the covering loop in these thiolases. The covering loop (residues 143–163) in PcaF is elongated (compared to the others) and has a narrower base. The loop comprises of both hydrophobic and hydrophilic residues and many of them are bulky amino acids (Fig. 6a). Comparison shows that the covering loop adopts a conformation tailored according to the ligand. For instance, in PcaF the natural substrate is β-Ketoadipyl CoA which has a linear acyl chain with two oxygen atoms at their tail. Thus, a covering loop that is long, narrow and amphiphilic, seems to be a perfect fit for the natural substrate of PcaF. On the other hand, the Mtb-thiolase is a steroid binding thiolase with the covering loop ranging from 128 to 148 amino acids. It is also amphiphilic as in PcaF but adopts a wider skeleton so that it can bind the bulky steroid substrate (Fig. 6c). On the contrary, Zr-thiolase has a small covering loop of eight amino acid residues (148–156) in length, which indicates the ability of the biosynthetic thiolases to bind smaller substrates (Fig. 6b). The analysis suggests that the size and nature of the covering loop plays a significant role by providing the ligand; the right space, volume and surroundings to bind and determining its substrate specificity in thiolases.

## 3 Discussion

**Fig. 7:**
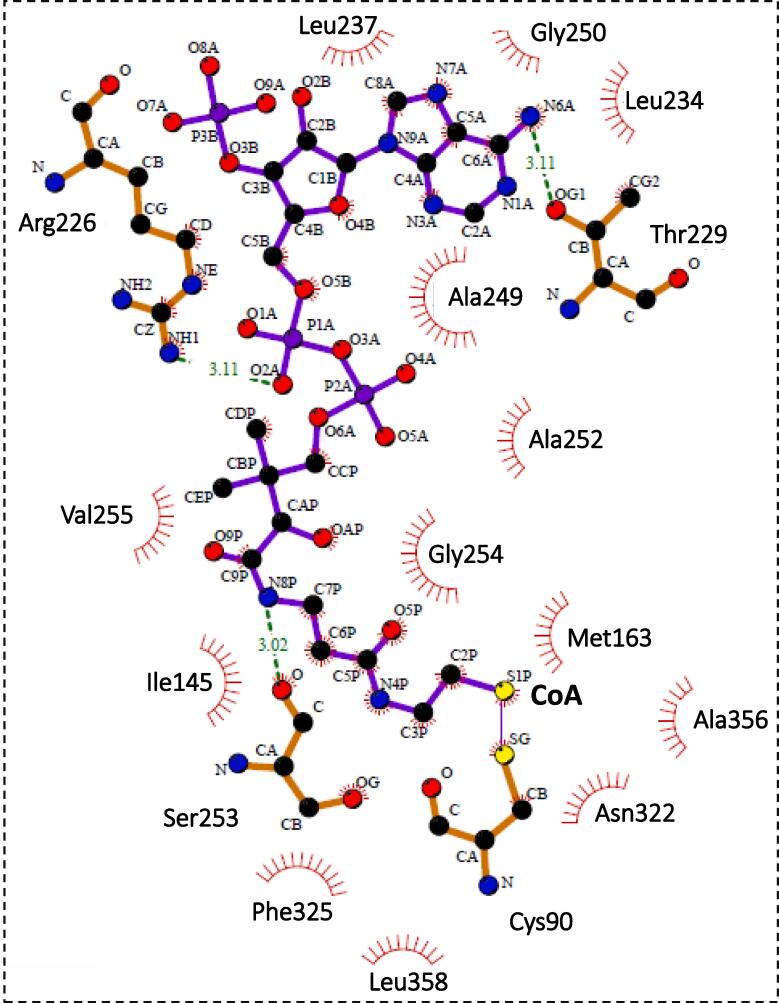
Schematic drawing of types of interaction of the CoA generated using LIGPLOT (Wallace et al., 1995). Covalent binding of CoA with C90 in A-mutant (H356A) - CoA complex was analysed by LIGPLOT and are presented in the software drawing. Disulphide bond between Sγ atom of C90 and the thiol group (-SH atom) of the CoA is represented as a thin cyan line. Hydrogen bonds are depicted with dashed line and hydrophobic interactions are shown as arcs.

The comparison of the degradative thiolase PcaF with the Zr-thiolase highlights the striking structure-based differences that assist in distinguishing these two classes of thiolases. The catalytic triad of the degradative thiolase and biosynthetic thiolase are identical and structural adopt a similar orientation. Whereas, the active site architecture residues that line the wall of the cavity and the tunnel length are starkly different. In the degradative thiolases, the active site architecture residues are less conserved as they bind to a wider variety of substrates. In comparison the biosynthetic thiolases which have a restricted binding capacity of four carbon chain acyl CoA substrate, with limited substrate scope. Thus, the biosynthetic thiolases comprise of highly conserved active site architecture residues. This indicates that even though the catalytic residues are alike, there is a global difference in the amino acid residues of the active site architecture, and this is mostly likely the driving forces of thiolase reaction in the biosynthetic or degradative directions. CoA and acyl CoAs are lengthy substrates and need tethering points to hold them in the correct configuration. The biosynthetic and degradative thiolases employ different anchoring residues to bind their substrates. In the Zr-thiolases, H157 acts as one of the anchoring residues and is conserved across the biosynthetic thiolases, but in the case of degradative thiolases, the residue at this position varies, implying that this position does not participate in substrate selection. In PcaF, H356 plays a central role in anchoring of the substrate, mutation of this residue leads to covalent locking of the reaction center. H356 also helps in retaining the active site architecture by maintaining the M163 and the R65 in correct conformation, these residues in turn are involved in anchoring the CoA in the optimal orientation. H356A mutation results in alternate conformation of Met 163 and causes the binding of CoA in an unusual orientation, exposing the thiol bond to the active site residue C90 instead of Cα and Cβ of 3-ketoacyl CoA consequently, covalently locking the active site (Fig. 7). Additionally, the alternate conformation of M163 provide ample space for R65 to adopt double conformation**.** However, a similar histidine mutation in Zr-thiolase (PDB code: 2WL4) exhibits no change in the side chain conformation of M157 and Q64 (equivalent to M163 and R65) residues. Thus, H356 of the catalytic triad has an additional role in anchoring the CoA to the active site and preserving the functionality of the thiolase. This further emphasizes the difference of the active site architectures and the role of this conserved histidine residue of the catalytic triad in the degradative and biosynthetic thiolases.

We investigated two possible causes of covalent locking in the A- and AA-mutant. Firstly, we perform X-ray radiation damage analysis and checked if the thioester bond of the ligand is broken due to X-ray radiation damage, causing the sulfur atom of the ligand to attacks the Sγ of C90 residue to form a covalent bond. However, this possibility seems to be less likely as the radiation damage analysis indicates breakage of the co-valent bonds between C90 and CoA, which means that the formation of covalent bond already took place during the soaking experiment prior to cryo-cooling of the protein crystals. This statement is further strengthened by the intact mass spectrometry experiment which were carried out at pH 7.5. The covalent modification of the A- or AA-mutants was observed in presence of the CoA derivatives (acetoacetyl CoA, hexanoyl CoA, octanoyl CoA).

The second possibility is that the C90 residue in A- and AA-mutant is deprotonated and acts as a nucleophile attacking the thioester bond of the ligand and forming the covalent bond with CoA part of the ligand. If this is the case, then the question is how C90 act as nucleophile in the pH range 7.5 to 8.0 (in which the intact mass spectrometry experiment and crystallization were carried out) when the pKa of the side chain of cysteine is ~ 8.3. It can be assumed that the pKa of the C90 may be lower than 8.3. It should be emphasized that, even if the C90 was active, the covalent locking would not have taken place if the H356 residue were present as it would had caused a steric hindrance to the C2P-S1P bond of CoA. This emphasize a role of H356 in allowing the ligand to bind in correct orientation, thus acting as “tethering point”.

Another striking observation is the presence of a substantially longer tunnel in the degradative thiolase PcaF compared to the Zr-thiolase. This longer tunnel allows PcaF to bind lengthier acyl chain CoA compounds, demonstrated by the ligand complexes of Hex-CoA/Oct-CoA. Both the HAL and OAL moieties interacts with the covering loop. Further superimposition of the degradative and biosynthetic tunnel indicates the role of the covering loop in determining tunnel length. The covering loop in biosynthetic thiolases is smaller which is found to restrict the length of the tunnel, unlike in the degradative thiolase PcaF where it is narrow and long (Fig. S8). Comparing the degradative thiolases PcaF, Mtb-thiolase and the biosynthetic thiolase Zr-thiolase has aided in understanding the importance of the covering loop in thiolases. In PcaF, the covering loop is lengthened and amphiphilic in nature which is pinched at the base causing the tunnel to be restrained breadthwise. Whereas, in Mtb-thiolase to accommodate steroid groups which also requires a wide comparable circular loop of mixed amino acids, the biosynthetic thiolase has a smaller wider sized loop. Comparison clearly shows that the covering loop length and its amino acid type determine the substrate size and nature. The covering loop is the distinctive feature which plays pivotal role in determining the degradative and biosynthetic classes.

In this study, we have demonstrated importance of the residues lining the active site in distinguishing the two class of thiolases. We have also shown additional function of H356 residue in maintaining the architecture in PcaF and difference in CoA binding between the two classes. Furthermore, we have provided structural insight into substrate adaptability and binding of longer chain substrate in degradative thiolases. This knowledge can be used to modify the tunnel to allow binding of specific type of substrates. Overall, our study has presented valuable structural information that could be applied to classify uncharacterized thiolases into degradative and biosynthetic type.

## 4 Materials and methods

### 4.1 Cloning, expression and purification

The clone of the PcaF in the pSGC-His vector with N-terminal His-tag including a TEV cleavage site was kindly provided by the New York Structural Genomics Research Consortium (NYSGRC). The PcaF construct was used as a template to make the following site mutations: C90S, H356A (A-mutant), and C90A. The construct of C90S and H356A was used as a template to make the double mutations H356A-C90S (AS-mutant), H356A-C386A (AA-mutant). All the mutants were made by site-directed mutagenesis by employing the “site-directed mutagenesis kit” from Kapa Biosystems (Kapa Biosystems). The native protein and the mutants were transformed in BL21 (DE3) pLysS cells and were plated onto chloramphenicol and kanamycin (concentrations 30 μg/mL and 35 μg/mL respectively) plates followed by 16 h incubation at 37⁰C. Colonies obtained in the above plates were inoculated into 5 mL LB pre-inoculum broth with the same concentration of chloramphenicol and kanamycin. Later, the 5 mL LB broth was transferred to the large-scale LB-kanamycin-chloramphenicol culture media which was then grown at 30⁰C by shaking it at 250 rpm. When the OD of the culture at 600 nm reached ~0.7, the culture was induced by 0.5 mM isopropyl-β-D- thiogalactopyranoside (IPTG) and the growth was continued at a reduced temperature of 25⁰C for 8 h. Afterward the cells were harvested by centrifugation at 4000 rpm for 30 min and purified using Ni-NTA resin by standard His-tagged affinity purification protocol involving lysis buffer (50 mM Tris-HCl buffer, pH 7.5; 2 mM Imidazole; 200 mM NaCl, 5 mM β-Mercaptoethanol), wash buffer (50 mM Tris-HCl buffer, pH 7.5; 30 mM Imidazole; 200 mM NaCl), and elution buffer (50 mM Tris-HCl buffer, pH 7.5; 350 mM Imidazole; 100 mM NaCl). The eluted fractions were desalted using an Econo-Pac 10 DG (Bio-Rad, CA, USA) column that was pre-equilibrated with a desalting buffer containing 25 mM Tris-HCl buffer, pH 7.5; 80 mM NaCl, 5% glycerol, and 0.5 mM DTT. The desalted protein fractions were pooled and concentrated up to 12.5 mg/ml, as determined by the Bradford assay using Bovine Serum Albumin (BSA) as a standard. The purity of the protein was verified by running a 10% SDS-PAGE followed by Coomassie Blue staining. The fractions were then flash-frozen in liquid N_2_ and stored at − 80 °C until they were used.

### 4.2 Crystallization, ligand soaking and data collection

Several commercially available crystallization screens were employed for initial crystallization screening, within a week crystals appeared in condition number 20 of the JCSG Suite [0.2 M MgCl2, 10% w/v PEG 8000, 0.1 M Tris (pH7.0)] and condition number 45 of the PACT Suite [0.2 M LiCl, 20% PEG 6000, 0.1 M Tris (pH-8.0). The crystallization trials were performed using hanging-drop vapor-diffusion method in Hampton 24-well plates. Based on initial x-ray characterization of crystals grown from the above conditions, the condition number 45 of the PACT Suite was further optimized.

For the apo as well as the mutant form of the protein, 1 μL protein solution mixed with 1 μL reservoir solution and was equilibrated with 500 μL reservoir solution. All the crystallization experiments were carried out at room temperature. Temperature of the crystallization plate was maintained in a temperature-controlled cabinet at 20 °C. Two different crystal forms were obtained from the same crystallization condition. The first crystal form appeared after 48 h of setting up crystal trays diffracted poorly and belonged to C2 space group with 16 molecules in an asymmetric unit. The second crystal form took three weeks to grow and corresponds to P2_1_2_1_2_1_ space group with 4 molecules in asymmetric unit. The orthorhombic form was further used for elucidation of native and ligand-complex structures. The native protein as well as the single and double mutant proteins (H356A (A-mutant), H356A-C90S (AS-mutant), H356A-C386A (AA-mutant)) were soaked with various substrates: Coenzyme A, Acetyl CoA, Acetoacetyl CoA, Hexanoyl CoA, Octanoyl CoA, Decanoyl CoA. The crystals were soaked for 20 s, 40 s, 1 min or 5 min. A single crystal of each ligand-complex was cryoprotected with 20% (*v*/*v*) ethylene glycol (prepared using mother liquor) including 3 mM of ligand prior to data collection.

X-ray diffraction experiments were performed at the micro-focus beamline (MX2) of the Australian Synchrotron (Aragao et al., 2018). The crystals were flash cooled in liquid nitrogen and transferred to a stream of nitrogen gas at 100 K. X-ray data were collected at a wavelength of 0.9537 Å using an EIGER-16 M detector with 0.1° oscillation and 0.1 s exposure of 0% attenuated beam per frame. 1800 frames of each data set were collected in 18 sec. Altogether 18 datasets were recorded in the resolution range of 2.56 to 1.37 Å. The data were indexed and integrated with XDS (Kabsch, 2010) and scaled using AIMLESS (Evans and Murshudov, 2013). Details of the data collection from the apo PcaF, A-mutant-CoA complex, A-mutant-Hex-CoA complex and AS-mutant-Oct-CoA crystals and its statistics from subsequent processing are presented in Table 1.

### 4.3 Structure determination and refinement

The Apo and Ligand–complex structures were solved using the MR protocol of the software pipeline Auto-Rickshaw (Panjikar et al., 2005, Panjikar et al., 2009). Structure of tt0182 (a putative acetyl-CoA acetyltransferase) from *Thermus thermophilus* HB8 (PDB code: 1ULQ) was used as a template for solving the structure of the Apo PcaF. The two molecules of the thiolase (which forms a dimer) were used as the search model for molecular replacement. Within the software pipeline, MR was performed using the program using MOLREP (Vagin and Teplyakov, 2000) and rigid-body, positional and B-factor refinement were carried out using the program CNS to 3.0 Å resolution. The structure was further refined using REFMAC5 (Murshudov et al., 2011) to its maximum resolution. Density modification was carried out using PIRATE (Collaborative Computational Project Number 4, 1994) and model building was carried out using ARP/wARP (Langer et al., 2008). The resulting model was further improved by rebuilding in the graphics program COOT (Emsley et al., 2010). Refinement was performed using REFMAC5 (Murshudov et al., 2011).

The native PcaF structure was used for solving all ligand complexes using MR protocol of Auto-Rickshaw (Panjikar et al., 2005, Panjikar et al., 2009). The resulting model for each complex, was refined in REFMAC5 (Murshudov et al., 2011) without any use of non-crystallographic symmetry (NCS) restrain. Water molecules were added in the difference density. The resulting map of each ligand complex was analyzed and difference density for the ligand was located using the graphic program COOT. The CIF file for the ligand was prepared using the program ACEDRG (Collaborative Computational Project Number 4, 1994) and used for the ligand building in COOT. The refinement statistics are shown in Table 1. The quality of the final model was validated with MolProbity (Chen et al., 2010).

### 4.4 Structure analysis

Crystal structures of the degradative thiolases from *Mycobacterium tuberculosis* (Mtb-thiolase) and the biosynthetic thiolase from *Zoogleria ramigera* (Zr-thiolase) were used in this study for structural comparisons. In particular, Mtb-thiolase in complex with steroid (PBD code: 4UBT) and four structures from the Zr-thiolase; the apo protein (PDB code: 1DLU), Zr-thiolase complexed with CoA (PDB code: 1DLV), C89A mutant of Zr-thiolase complexed with CoA (PDB code: 2WL4) and H348A mutant of Zr-thiolase complexed with acetoacetyl CoA (PDB code: 1M1O). All structural superpositions were performed using the SSM protocol of COOT. All figures showing structural representation were prepared with PyMol (DeLano, 2002).

### 4.5 Channel analysis

Two online programs were used to identify the tunnel in the native structure of PcaF and to measure the geometry of the tunnel. CAVER 3.0.1 (Chovancova et al., 2012) program was used with its default parameters and with a shell radius of 3 Å in order to foretell the presence of tunnels in the protein. CASTp program (Tian et al., 2018) was used for calculations and prediction of surface accessibility as well as internal cavities of the protein structures. It was used to confirm as well as to measure area of the promising potential/cavity.

### 4.6 Sequence alignment

100 sequences each for biosynthetic and degradative thiolases were sought using Blastp in NCBI. PcaF sequence was used as a reference for degradative thiolases against which 100 sequences below 95% sequence identity were curated. A similar criterion was used for the 100 biosynthetic sequences where Zr-thiolase was used as the query sequence.

### 4.7 Intact mass spectrometry

The mass spectrometry was performed using the native protein and the AA mutant. The control mixture for the native protein consist of 0.25 M Tris-HCl buffer (pH 7.5), 1 mg/ml native protein. The ligand mixture for the native protein is same as control mixture but with 0.5 mM of acetoacetyl CoA. The mixture was incubated for 30 mins at 37 °C before the experiment.

The control mixture for the AA-mutant consist of 0.25 M Tris-HCl buffer (pH 7.5), 1 mg/ml AA-mutant. Three ligand mixture for the AA-mutant were prepared with each sample having different ligand of acetoacetyl CoA/hexanoyl CoA/Octanoyl CoA. All the protein–ligand sample consist of 0.25 M Tris-HCl buffer (pH 7.5), 1 mg/ml AA-mutant, and 0.5 mM of ligand. The three reaction mixtures were incubated for 30 mins at 37 °C. The reaction mixture then used for mass spectrometry. Protein samples were analysed by LC-MS using a quadrupole TOF mass spectrometer (MicroTOFq, Bruker Daltonics, Bremen, Germany) coupled online with a 1200 series capillary HPLC (Agilent technologies, Santa Clara, CA, USA). Samples injected onto a MabPac SEC-1 5um 300A 50x4mm (Thermo Scientific) column with 50% Acetonitrile 0.05%TFA, 0.05% FA at a flow rate of 50ul/minute. The protein is eluted with monitoring by UV detection at 254 nm. The eluant is nebulised and ionised using the Bruker electrospray source with a capillary voltage of 4500 V dry gas at 180 °C, flow rate of 4 l/minute and nebuliser gas pressure at 300 mbar. Low concentration Tune mix (Agilent technologies, Santa Clara, CA, USA) use directly infused at the end of the run to calibrate the spectrum post acquisition. The spectra were extracted and deconvoluted using Data explorer software version 3.4 build 192 (Bruker Daltonics, Bremen, Germany).

### 4.8 Radiation damage calculation of ligand complex

180° of X-ray dataset were collected from the ligand complex datasets as described in “Crystallization, ligand soaking and data collection” section. In order to analyze, radiation damage, first 90° and last 90° of datasets were processed in XDS and the resulting datasets is termed as “before” and “after” dataset respectively. Overall completeness of each dataset was still about 90% due to primitive orthorhombic space group. The refined ligand complex structure was used for model phase. Isomorphous difference Fourier map was created by using the program ANODE (Thorn and Sheldrick, 2011) with the model-phase and the structure factor (F _before_ − F _after_) created using the program SHELXC (Sheldrick et al., 2001) with ‘RIP’ option. The resulting map was used for radiation damage analysis along with the structure.

### 4.9 Enzymatic studies

The degradative activity for PcaF was measured and the kinetic parameters for the native protein calculated. Thiolase thiolytic cleavage activity was performing using the modified Mg^+2^ method (Ithayaraja et al., 2016, Stern, 1956). The reaction mixture had 70 μM CoASH and variable amount of (10–120 μM) Acetoacetyl-CoA in 0.1 M TrisHCl (pH 8.3) buffer containing 25 mM MgCI_2_. The loss of acetoacetic CoA enolate chromophore was followed at 303 nm after 15 min of incubation at 37 °C. Kinetics study of the native PcaF was followed by activity assessment of the three mutants: H356A (A-mutant), C90S-H356A (AS-mutant), H356A-386A (AA-mutant).

### 4.10 Accession numbers

The atomic co-ordinates and experimental structure factors were deposited in the Protein Data Bank under accession code 6PCA (Apo structure), 6PCB (A-mutant CoA complex), 6PCC (A-mutant-Hex-CoA complex) and 6PCD (AS-mutant-Oct-CoA).

## Author contributions

S. B. has contributed in methodology, investigation, formal analysis, visualization and writing. S. P. has contributed in conceptualization, methodology, visualization, writing and supervision. R. A. has contributed in visualization, writing and supervision. D.S. has contributed in investigation of mass spectrometry.

## Declaration of Competing Interest

The authors declare that they have no known competing financial interests or personal relationships that could have appeared to influence the work reported in this paper.
