# Supplementary material for: Structural basis for differentiation between two classes of thiolase: Degradative vs biosynthetic thiolase
Source: J Struct Biol X. 2020 Jan 3;4:100018. doi: 10.1016/j.yjsbx.2019.100018 (PMC7337054; doi:10.1016/j.yjsbx.2019.100018)
Supplement: Supplementary data 1 [file mmc1.docx]

# Supplementary Information


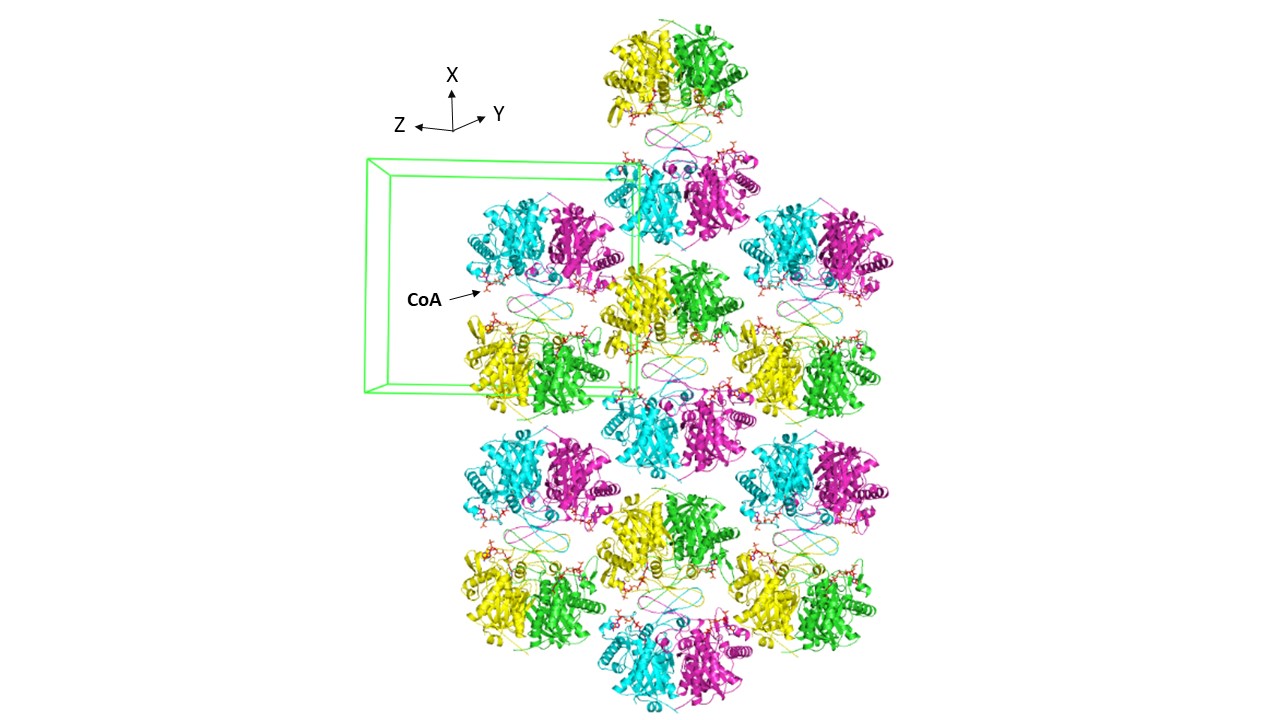


**Fig. S1:** Packing diagram for the H356A (A-mutant) crystal in presence of CoA. Unit cell shown as green inline and cell axis direction in black.


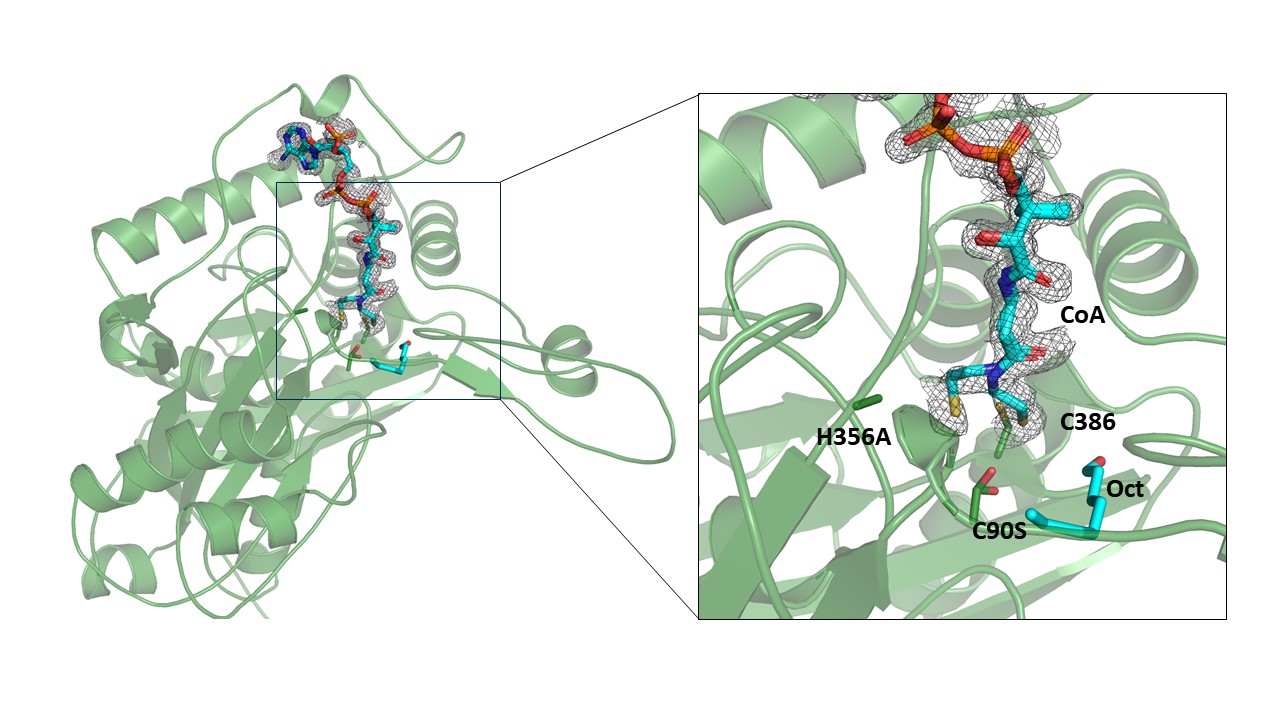


***Fig. S2:*** *AS-mutant complexed with Octanoyl CoA. The thiol group of CoA in double conformation.* Simulated annealing omit map *for the CoA contoured at 3σ. AS-mutant is marked with H356A and C90S, in which C90S also adopts double conformation.*


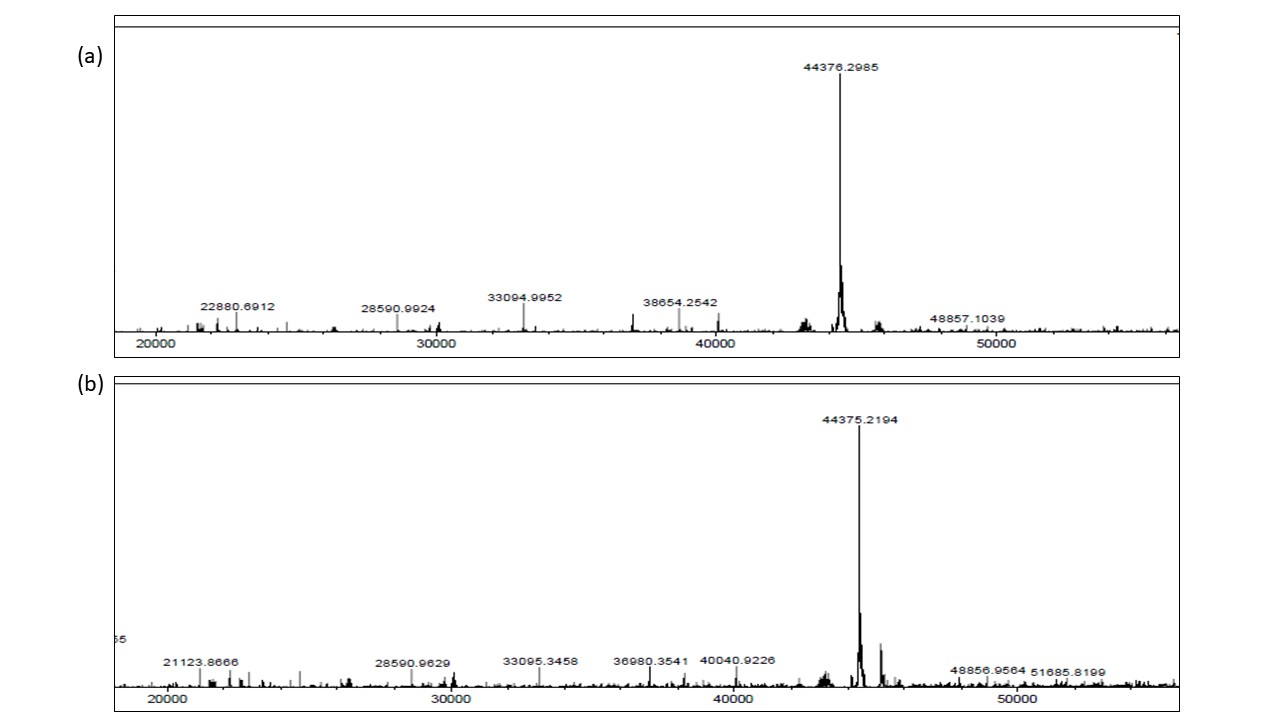


**Fig. S3**: The intact mass spectrometry spectrum for (a) the Apo-native (control) protein with a peak at 44376.3 Da. (b) the Apo-acetoacetyl CoA complex which has also a peak at 44376.3 Da.

***
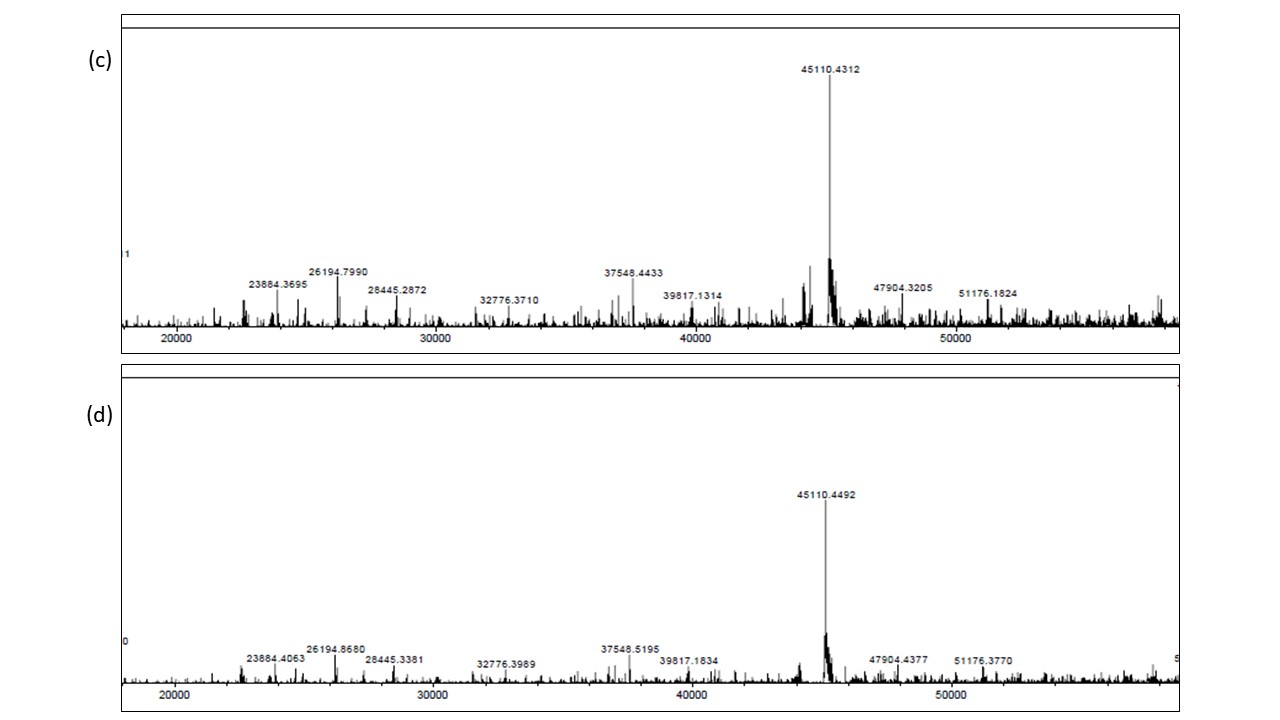

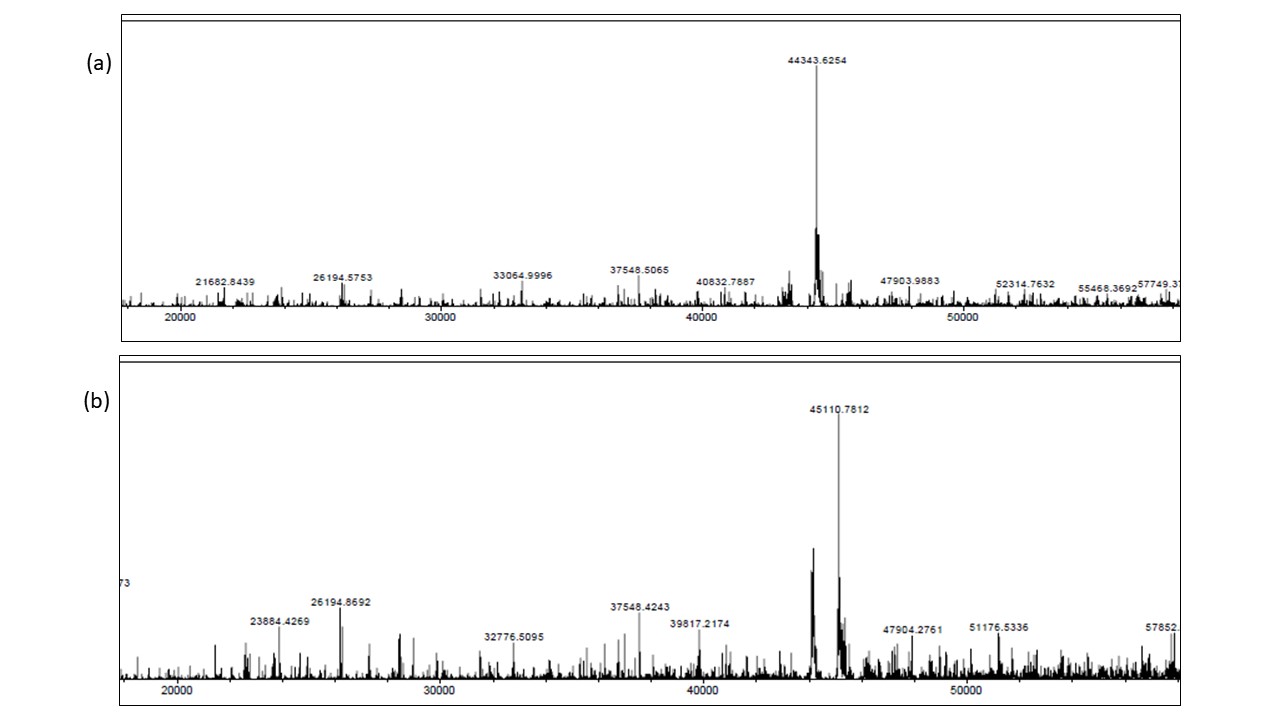
***

***Fig. S4****: The intact mass spectrometry spectrum for (a) the AA-mutant (control) protein, a peak at 44343.6 Da, (b) the AA-acetoacetyl CoA complex, a peak at 45110.8 Da, (c) the AA-hexanoyl CoA complex, a peak at 45110.4 Da (d) the AA-octanoyl CoA complex, a peak at 45110.4 Da.*


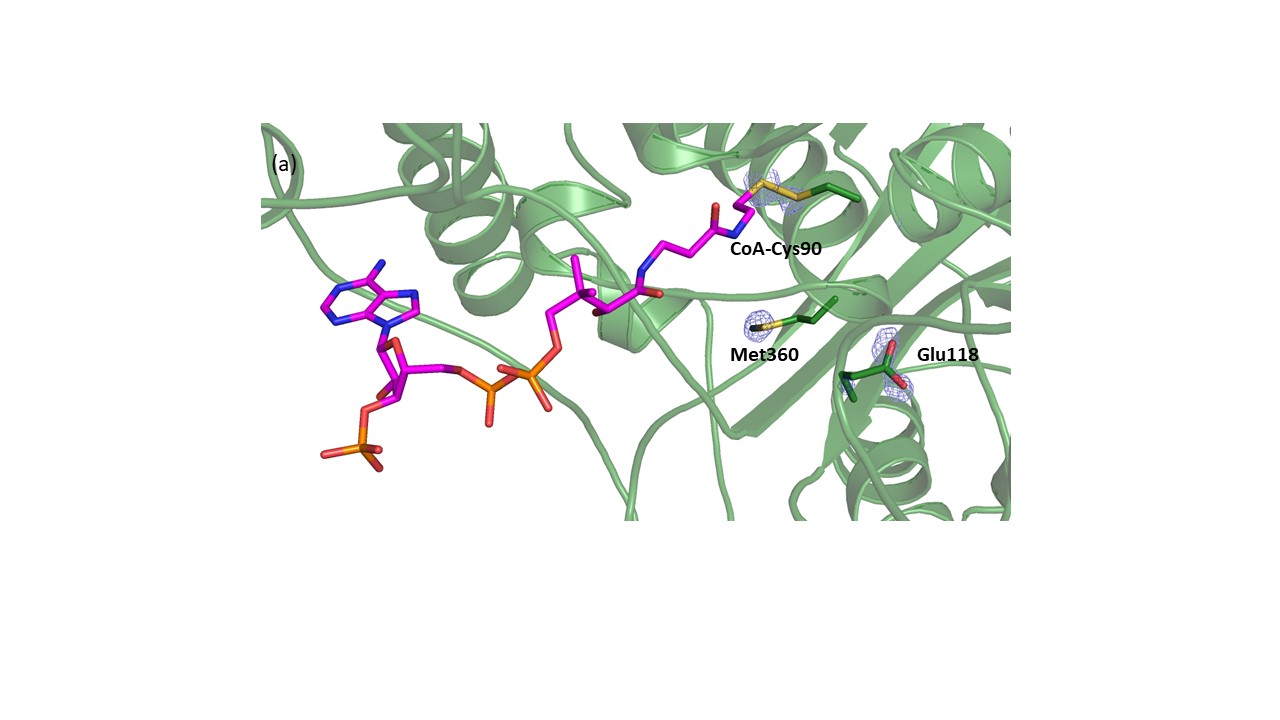


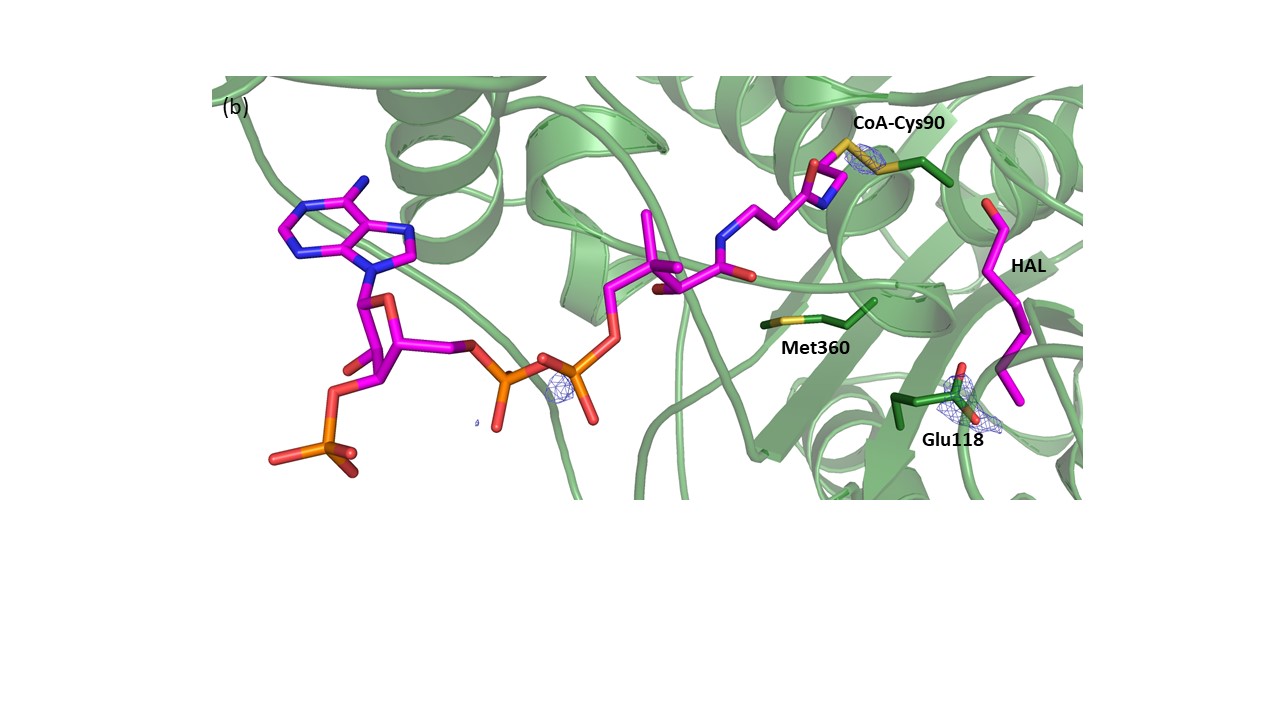


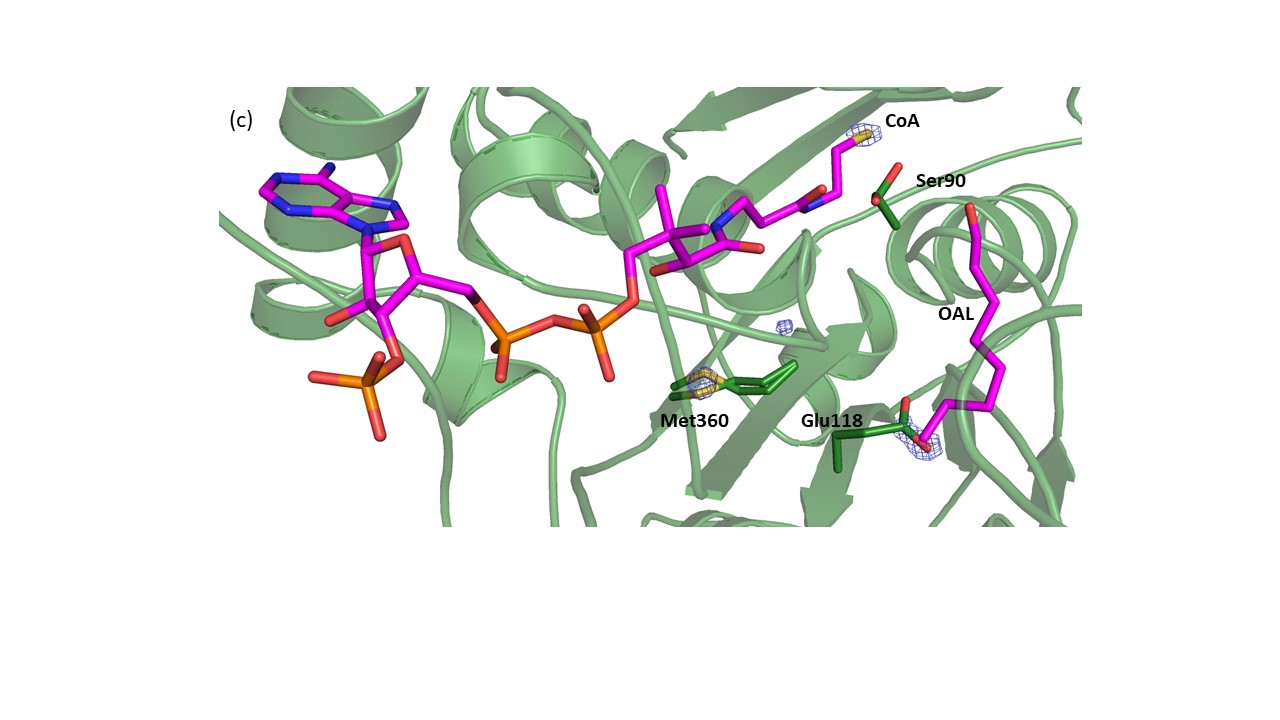


**Fig. S5:** Radiation damage analysis **(a)** A-mutant complex with CoA **(b)** A-mutant complex with hexanoyl CoA **(c)** AS-mutant complex with octanoyl. Isomorphous difference Fourier map (f_before_ – f_after_, model_phase) for each structure has been shown at above 3σ.


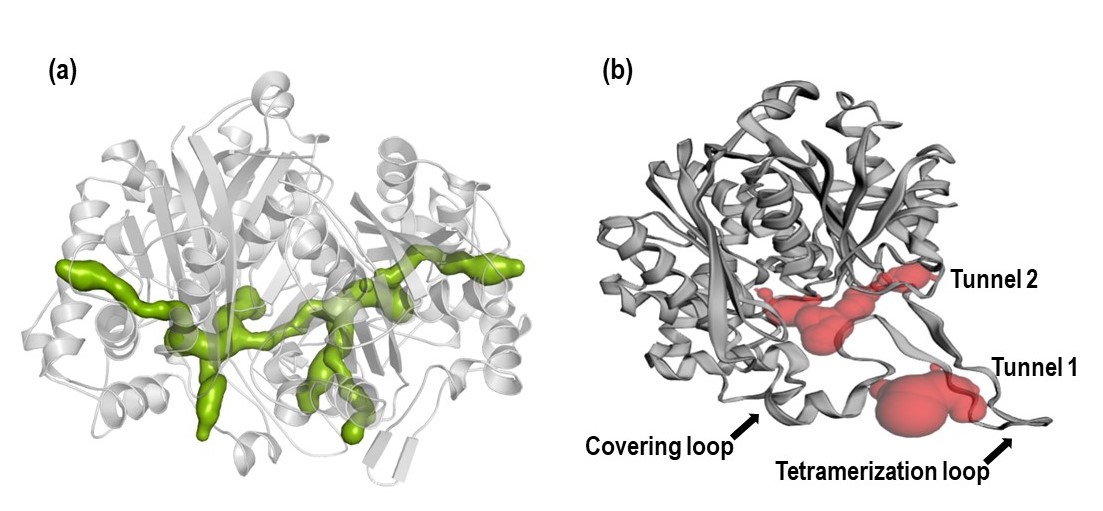
 ***Fig. S6****: (a) The interconnected tunnels in dimeric PcaF (calculated using CAVER [1] ) (b) The two tunnels (Tunnel 1 and Tunnel 2) in the monomeric PcaF are shown as red surface (calculated using CASTp [2]). Tunnel 1 is located at the tetramerization loop while tunnel 2 located at the covering loop of the PcaF.*


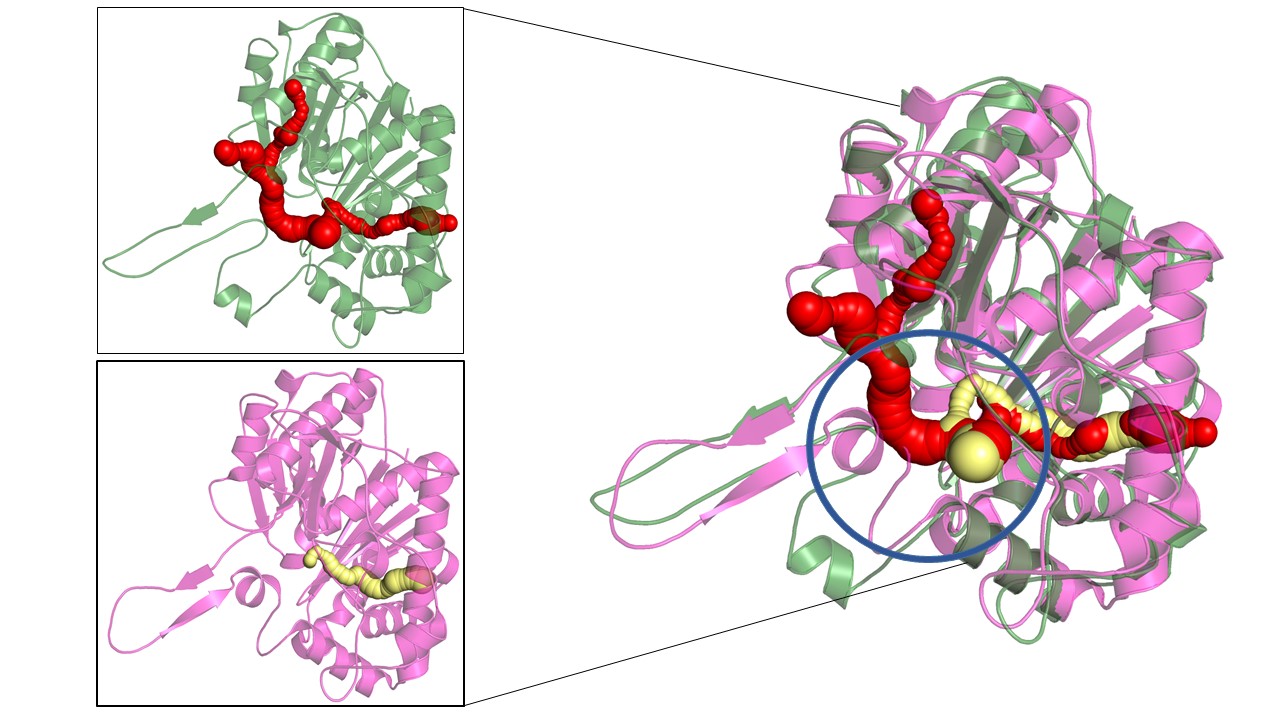


***Fig. S7****: Tunnel comparison in the Zr-thiolase (PDB code: 1DLU) and the degradative thiolase PcaF structures. The structure of apo PcaF and its long tunnel are shown in green and red respectively. The tunnel of the biosynthetic thiolase is shown in yellow. Superimposition of the both structures shows that the biosynthetic covering loop block the degradative tunnel and makes it shorter indicating importance of the covering loop in tunnel formation.*


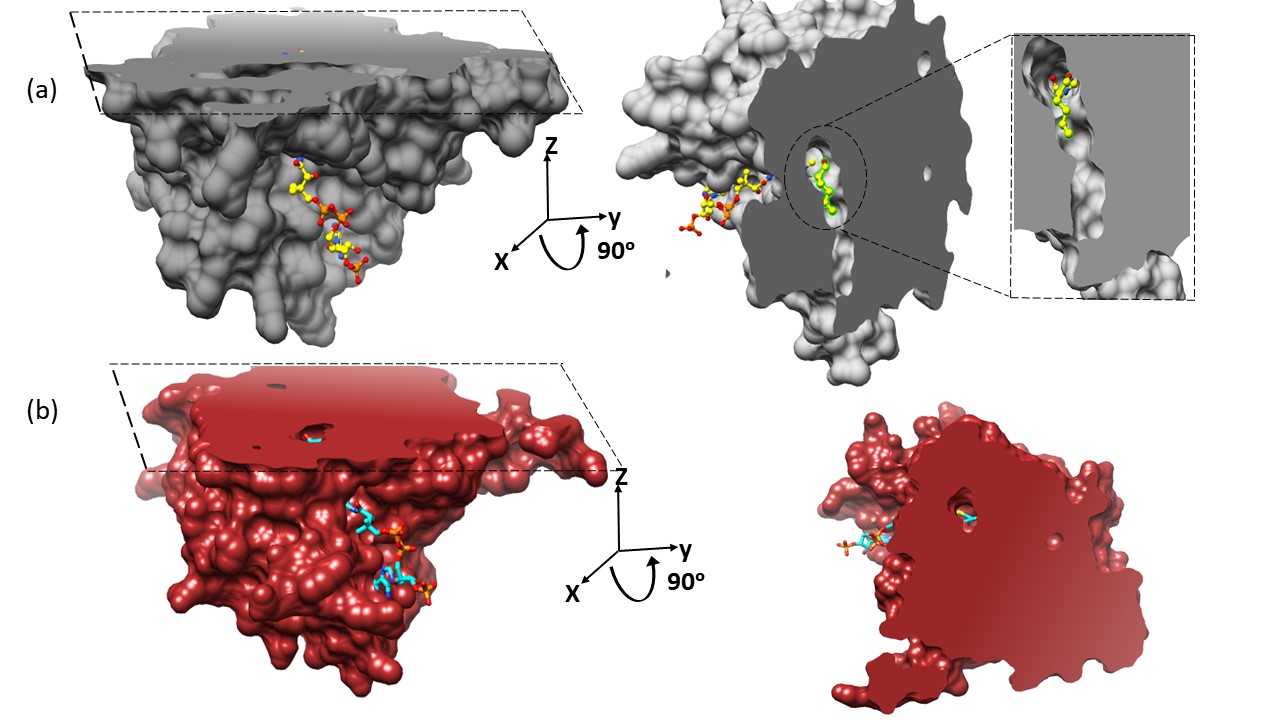


***Fig. S8****: The comparison of the tunnel in Zr-thiolase (PDB code:1M1O) and degradative thiolase PcaF (a) The structure of A-mutant (H356A) complexed with Hexanoyl CoA is used here. The PcaF protein is clipped through to visualize the long tail acyl binding tunnel. The bound hexanal and CoA shown in yellow does not fully occupy the tunnel (b) The tunnel of biosynthetic thiolase is studied using the C89A mutant complexed with Acetoacetyl-CoA (PDB code:1M1O). The clipped structure highlights the absence of a long tunnel in biosynthetic thiolase.*

**Table S1**: RSCC values for the ligands found in 6PCB (A-mutant CoA complex)

| LIGAND | CHAIN | RSCC |
| --- | --- | --- |
| Coenzyme A (COA) | A | 0.87 |
| Coenzyme A (COA) | B | 0.85 |
| Coenzyme A (COA) | C | 0.87 |
| Coenzyme A (COA) | D | 0.88 |
| Glycerol (GOL) | A | 0.98 |
| Glycerol (GOL) | B | 0.97 |
| Glycerol (GOL) | C | 0.96 |
| Glycerol (GOL) | D | 0.96 |

**Table S2**: RSCC values for the ligands found in 6PCC (A-mutant-Hex-CoA complex)

| LIGAND | CHAIN | RSCC |
| --- | --- | --- |
| Coenzyme A (COA) | A | 0.90 |
| Coenzyme A (COA) | B | 0.88 |
| Coenzyme A (COA) | C | 0.90 |
| Coenzyme A (COA) | D | 0.88 |
| Hexanal (O8Y) | A | 0.91 |
| Hexanal (O8Y) | B | 0.93 |
| Hexanal (O8Y) | C | 0.96 |
| Hexanal (O8Y) | D | 0.92 |
| Glycerol (GOL) | A | 0.95 |
| Glycerol (GOL) | B | 0.94 |
| Glycerol (GOL) | C | 0.95 |
| Glycerol (GOL) | D | 0.96 |

**Table S3**: RSCC values for the ligands found in6PCD (AS-mutant-Oct-CoA).

| LIGAND | CHAIN | RSCC |
| --- | --- | --- |
| Coenzyme A (COA) | A | 0.71 |
| Coenzyme A (COA) | D | 0.69 |
| Octanal (OYA) | A | 0.82 |
| Octanal (OYA) | B | 0.86 |
| Octanal (OYA) | D | 0.82 |
| Glycerol (GOL) | A | 0.98 |
| Glycerol (GOL) | B | 0.98 |
| Glycerol (GOL) | C | 0.98 |
| Glycerol (GOL) | C | 0.96 |
| Glycerol (GOL) | D | 0.97 |

# References

[1] E. Chovancova, A. Pavelka, P. Benes, O. Strnad, J. Brezovsky, B. Kozlikova, A. Gora, V. Sustr, M. Klvana, P. Medek, L. Biedermannova, J. Sochor, J. Damborsky, CAVER 3.0: A Tool for the Analysis of Transport Pathways in Dynamic Protein Structures, PLoS Comput. Biol. 8 (2012) 23–30. doi:10.1371/journal.pcbi.1002708.

[2] W. Tian, C. Chen, X. Lei, J. Zhao, J. Liang, CASTp 3.0: computed atlas of surface topography of proteins., Nucleic Acids Res. 46 (2018) W363–W367. doi:10.1093/nar/gky473.
